# Evidence of IL-17, IP-10, and IL-10 involvement in multiple-organ dysfunction and IL-17 pathway in acute renal failure associated to *Plasmodium falciparum* malaria

**DOI:** 10.1186/s12967-015-0731-6

**Published:** 2015-11-24

**Authors:** Fabien Herbert, Nicolas Tchitchek, Devendra Bansal, Julien Jacques, Sulabha Pathak, Christophe Bécavin, Constantin Fesel, Esther Dalko, Pierre-André Cazenave, Cristian Preda, Balachandran Ravindran, Shobhona Sharma, Bidyut Das, Sylviane Pied

**Affiliations:** CIIL-Center for Infection and Immunity of Lille, Team 04: Basic and Clinical Immunology of Parasitic Diseases, INSERM U1019, CNRS UMR 8204, Univ Lille Nord de France, Institut Pasteur de Lille, 1, rue du Prof Calmette, 59019 Lille Cedex, France; CEA, DSV/iMETI, Immunology of Viral Infections and Autoimmune Diseases Research Unit, UMR1184, IDMIT Infrastructure, Fontenay-aux-Roses, France; LPP, Laboratoire Paul Painlevé, INRIA Lille, Nord Europe, MODAL, Villeneuve-d’Ascq, France; Department Biological Sciences, Tata Institute of Fundamental Research, Mumbai, India; Unité des Interactions Bactéries-Cellules, Institut Pasteur, 75015 Paris, France; Instituto Gulbenkian de Ciencia, Oeiras, Portugal; Immunologie, Immunopathologie, Immunothérapie, UPMC/CNRS UMR 7211, Paris, France; Institute of Life Sciences, Bhubaneswar, Odisha India; SCB Medical College, Cuttack, Odisha India

**Keywords:** *Plasmodium falciparum*, Severe malaria, Cerebral malaria, Acute-renal failure, Malaria associated organ failure, IL-17, Cytokines, Biomarkers

## Abstract

**Background:**

*Plasmodium falciparum* malaria in India is characterized by high rates of severe disease, with multiple organ dysfunction (MOD)—mainly associated with acute renal failure (ARF)—and increased mortality. The objective of this study is to identify cytokine signatures differentiating severe malaria patients with MOD, cerebral malaria (CM), and cerebral malaria with MOD (CM-MOD) in India. We have previously shown that two cytokines clusters differentiated CM from mild malaria in Maharashtra. Hence, we also aimed to determine if these cytokines could discriminate malaria subphenotypes in Odisha.

**Methods:**

*P. falciparum* malaria patients from the SCB Medical College Cuttack in the Odisha state in India were enrolled along with three sets of controls: healthy individuals, patients with sepsis and encephalitis (n = 222). We determined plasma concentrations of pro- and anti-inflammatory cytokines and chemokines for all individuals using a multiplex assay. We then used an ensemble of statistical analytical methods to ascertain whether particular sets of cytokines/chemokines were predictors of severity or signatures of a disease category.

**Results:**

Of the 26 cytokines/chemokines tested, 19 increased significantly during malaria and clearly distinguished malaria patients from controls, as well as sepsis and encephalitis patients. High amounts of IL-17, IP-10, and IL-10 predicted MOD, decreased IL-17 and MIP-1α segregated CM-MOD from MOD, and increased IL-12p40 differentiated CM from CM-MOD. Most severe malaria patients with ARF exhibited high levels of IL-17.

**Conclusion:**

We report distinct differences in cytokine production correlating with malarial disease severity in Odisha and Maharashtra populations in India. We show that CM, CM-MOD and MOD are clearly distinct malaria-associated pathologies. High amounts of IL-17, IP-10, and IL-10 were predictors of MOD; decreased IL-17 and MIP-1α separated CM-MOD from MOD; and increased IL-12p40 differentiated CM from CM-MOD. Data also suggest that the IL-17 pathway may contribute to malaria pathogenesis via different regulatory mechanisms and may represent an interesting target to mitigate the pathological processes in malaria-associated ARF.

**Electronic supplementary material:**

The online version of this article (doi:10.1186/s12967-015-0731-6) contains supplementary material, which is available to authorized users.

## Background

Malaria, caused by *Plasmodium* spp., results in 207 million cases worldwide and 627,000 deaths annually [1]. *P. falciparum* infection may lead to cerebral malaria (CM), a major contributor to malaria-associated mortality [2]. In India, the incidence of falciparum malaria is on the rise [3], and the clinical presentation of severe falciparum malaria has dramatically changed in the last decade, with a shift toward multiple complications [3]. The incidence of CM patients with MOD (multiple organ dysfunction) (CM-MOD), most commonly associated with acute renal failure (ARF), is increasing; mortality is mainly observed in patients with ARF [4, 5]. However, the underlying pathophysiology for this manifestation of malaria severity remains poorly understood.

Severe malarial pathology is the consequence of the interplay of detrimental factors of parasite- and host-origin during their interaction [6]. Parasite sequestration in organ microvessels, the underlying inflammatory process and a disturbed pro- and anti-inflammatory cytokine balance results in malaria pathology [7–10]. The literature on malaria as a systemic inflammatory disease is extensive. So far, several studies show that severe malaria is associated with increased TNF-α, IFN-γ, and IL-1β but decreased IL-10 and TGF-β [11–17]. However, increase pro-inflammatory cytokines (e.g., IFN-γ and TNF-α) were also associated with protection [18, 19]. Despite their complex network with multiple regulatory pathways, their changed expression patterns have potential as disease biomarkers. Several studies in human have identified cytokines profiles able to differentiate malaria clinical subphenotypes [20–22].

In a previous study done in Gondia, an endemic malaria region in the state of Maharashtra in India, we identified two cytokine clusters that could differentiate CM from mild malaria (MM) using a two-way coupled clustering approach [23]. Furthermore, linear discriminant analysis showed that IL-1β but not TNF-α contribute development of CM in Gondia. We concluded that this approach was suited to distinguishing severe malaria subphenotypes (e.g. CM and CM-MOD) in India. Currently, there is no prediction scoring system to prognosticate disease outcome in adult patients. Therefore, identification of biomarkers that are sensitive and specific to disease severity will be beneficial, especially as blood-based markers such as cytokines can be used in clinical trials.

The present study aims to determine whether cytokine clusters identified in Gondia could discriminate CM from other subphenotypes in Odisha [23]. So, we profiled plasma cytokine/chemokine levels in falciparum malaria patients from an area of Odisha with an Annual Parasite Index (API) >5 [24]. The state is falciparum malaria endemic, with manifestations including asymptomatic, MOD, CM and CM-MOD [4, 5]. Using multiple statistical tests, we identified differentially expressed cytokines and their contributions to the clinical malaria spectrum. We focused on cytokine signatures differentiating the MOD, CM, and CM-MOD categories. High amounts of IL-17, IP-10, and IL-10 were predictors of MOD; decreased IL-17 and MIP-1α separated CM-MOD from MOD; and increased IL-12p40 differentiated CM from CM-MOD. Our data indicate that IL-17 may be a critical factor in ARF but not CM.

## Methods

### Ethics statement

The Institutional Human Ethics Committee of S. C. B. Medical College, Cuttack and Institute Pasteur, Lille, approved the study. Blood and urine were collected after obtaining written informed consent from participants or, for comatose patients, the accompanying person.

### Study site, clinical definition of malaria, and sample collection

The study was conducted at SCB Medical College and Hospital, Cuttack, Odisha. More than 85 % cases in Odisha are due to *P. falciparum* [5]. Subjects (n = 222) from coastal district areas with an API of 6.6 [24] were enrolled from 2008 to 2010. Parasite presence was tested with RDK (SD Bio Standard Diagnostic, India) and thick and thin blood smears. Nested PCR was used to detect *P. falciparum*/*P. vivax* co-infection [25]. Only falciparum positive cases were included in the study. Clinical histories, detailed physical assessments, demographic profiles and informed consent from all patients were recorded at admission. Using the World Health Organization’s severity criteria [26], patients were categorized as following: (1) 37 patients with uncomplicated malaria (MM) had fever without complications and were positive for *P. falciparum*, (2) 53 patients with severe non cerebral malaria (SNCM) which had one of the several manifestations of severe malaria without cerebral involvement such as severe anemia (haemoglobin ≤ 5 g/dl), jaundice (serum bilirubin ≥ 3 mg/dl), acute renal failure (serum creatinine ≥ 3 mg/dl), acute respiratory distress (PaO_2_/FIO_2_ ≤ 200), shock (systolic BP ≤ 80 mmHg) and haemoglobinuria (dark red or black coloured urine positive for haemoglobin), (3) nine patients with multiple organ dysfunction (MOD) were patients diagnosed for the presence of two or more organ involvement like CNS, respiratory distress, renal failure and hepatic dysfunction (ALT/AST ≥3 times of normal, prolonged prothrombin and albuminaemia), (4) 83 patients with fever and altered sensorium, unarousable coma with Glasgow Coma Scale (GCS) of ≤10 were categorized as cerebral malaria (CM) after exclusion of other causes of encephalopathy such as encephalitis, meningitis and metabolic encephalopathy by biochemical investigations in the CSF. Among CM, 41 patients with another organ failure was further defined as patients with MOD (CM-MOD), (5) 21 healthy subjects from malaria-endemic areas (EC) were patient’s relatives and who had not suffered from a bout of malaria for at least 2 years, nor were they asymptomatic carriers. We also added two control groups, 10 severe sepsis (SEPT) and 9 viral encephalitis (ENC) admitted in the Department of Internal Medicine for treatment. All blood smears were checked for the presence of malarial parasites (Table 1).

**Table 1:** Demographic profiles of the patients infected with *P. falciparum* malaria and controls

Groups	Criterion	No. of patients (%)	Median age (range)	Sex (M/F)
EC	Healthy subjects from malaria-endemic areas	21 (9.46)	29 (17–52)	20/1
SEPT	Severe sepsis patients	10 (4.5)	39 (24–70)	5/5
ENC	Viral encephalitis patients	9 (4.06)	35 (13–72)	7/2
MM	Patients having fever without complications	37 (16.67)	28 (15–62)	25/12
SNCM	Patients without cerebral involvement, but with either: severe anemia^a^ or jaundice^b^, or ARF^c^, or acute respiratory distress^d^, or shock^e^ or haemoglobinuria	53 (23.87)	34 (15–65)	36/17
MOD	Patients showing involvement of two or more organs: CNS, respiratory distress, ARF, or hepatic dysfunction^f^	9 (4.06)	28 (16–55)	8/1
CM	Patients with fever and altered sensorium, unarousable coma with Glasgow Coma Scale of ≤10^g^	42 (18.91)	28 (15–65)	32/10
CM-MOD	CM patients with MOD	41 (18.47)	35 (15–70)	31/10
Total		222 (100 %)	30 (13–72)	164/58

*EC* endemic control, *SEPT* septicemia, *ENC* encephalitis, *MM* mild malaria, *SNCM* severe non-cerebral malaria, *MOD* multiple organ dysfunction, *CM* cerebral malaria, *CM-MOD* cerebral malaria with multiple organ dysfunction, *ARF* acute renal failure

^a^Haemoglobin ≤ 5 g/dl

^b^Serum bilirubin ≥ 3 mg/dl

^c^Serum creatinin ≥ 3 mg/dl

^d^PaO_2_/FIO_2_ ≤ 200

^e^Systolic BP ≤ 80 mmHg

^f^ALT/AST ≥3 times of normal, prolonged prothrombin time, and albuminaemia

^g^Categorized as CM after excluding other causes of encephalopathy, such as encephalitis, meningitis, and metabolic encephalopathy, by biochemical investigations of CSF

### Blood collection, diagnosis, and assessment of biological parameters

Peripheral venous blood (5 mL) was collected on day 0, before treatment. Plasma, obtained by centrifuging (4500*g*, 15 min), was stored at −80 °C. Participants underwent tests for complete blood count, renal and liver function, blood sugar, electrolytes, and lactates. Coagulation profile was obtained for patients with disseminated intravascular coagulation, viral markers for those with hepatopathy and jaundice, and arterial blood gas analysis for ones with respiratory distress syndrome.

### Cytokine measurements

Plasma concentrations of 26 cytokines: GM-CSF, Eotaxin, IL-1α, MCP-1, IL-10, MIP-1α, MIP-1β, IL-8, IL-6, TNF-α, IFN-α2, IL-7, G-CSF, IP-10, IL-3, IL-5, IL-15, TNF-β, IL-2, IL-13, IL-4, IL-12p70, IL-17, IFN-γ, IL-12p40, IL-1β were measured using Luminex multianalytic profiling (MILLIPLEX^®^ MAP human cytokine/chemokine—Premixed 26 Plex), according to manufacturer instructions (Millipore, USA). Cytokine detection (range 1–10,000 pg/mL) and quantification were performed using a Bio-Plex 200 system (Bio-Rad, USA). Samples were tested in duplicate. Data were analyzed using Bio-Plex Manager 5.0 software (Bio-Rad).

### Statistical methods

All analysis was conducted on log_10_-transformed data. Normality of the data was checked using the Shapiro–Wilk test. Differentially expressed cytokines were identified using the Student’s *t* test; p < 0.05 was considered significant. Cytokine expression heatmaps, and hierarchical clustering of cytokines and of the samples within each category, were generated based on the Euclidean metric and using R/Bioconductor. Multidimensional scaling (MDS) representation was generated using SVD-MDS [27]. MDS methods aim to represent the similarities and differences among high dimensionality objects into a space of a lower dimensions, generally in two or three dimensions for visualization purposes [28]. The Spearman coefficient of correlation was used to determine the association between cytokines. Linear Discriminant Analysis (LDA) was performed on log-transformed data from the Gondia study and the present study using macros and IgorPro software (version 3.16; WaveMetrics). Samples with incomplete information were rejected. No correction for multiple tests was applied to the p values.

R software’s glm function was combined with a stepwise selection procedure based on the Akaike information criterion for logistic regression analysis [29]. Such selection introduces, at each step, the cytokine yielding the best Akaike information criterion and removes non-significant cytokines (p > 0.05) [30]. The true positive rate (sensitivity) and true negative rate (specificity) were estimated for the obtained model; area under the Receiver Operating Characteristic curve was used to assess quality.

## Results

### Distributions of circulating cytokines/chemokines in clinical subphenotypes of falciparum malaria in Odisha patients

We quantified levels of GM-CSF, Eotaxin, IL-1α, MCP-1, IL-10, MIP-1α, MIP-1β, IL-8, IL-6, TNF-α, IFN-α2, IL-7, G-CSF, IP-10, IL-3, IL-5, IL-15, TNF-β, IL-2, IL-13, IL-4, IL-12p70, IL-17, IFN-γ, IL-12p40, IL-1β, by multiplex assay and generated an expression heatmap.

Although control groups (EC, SEPT, ENC) clustered together in the MDS representation, malaria subgroups, particularly severe forms, could not be clearly distinguished based on the whole set of cytokines (Additional file 1: Figure S1). Only the MOD group could be distinguished from the control groups. This suggests that the malarial inflammatory process is different from that of encephalitis and septicemia.

**Fig. 1:**
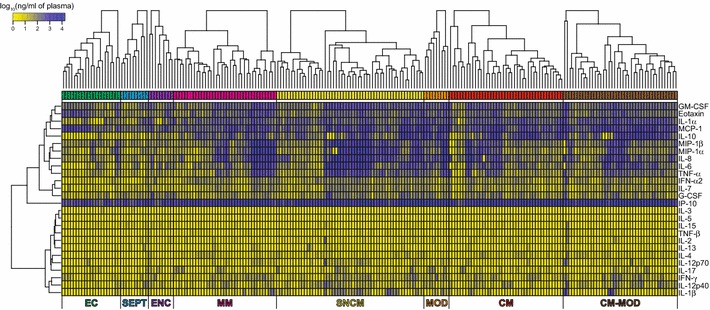
Heatmaps of cytokine profiles in malarial subgroups and controls. Heatmaps showing the log_10_-transformed cytokine expression values for each individual sample. Hierarchical clusters of cytokines are represented by a vertical dendrograms. In order to identify group sub-populations, hierarchical clusters within each biological condition are represented by horizontal dendrograms. Hierarchical clusters of cytokines and samples were created based on the Euclidean distance and using the complete linkage agglomeration method

We aimed then to identify groups of cytokines having similar expression patterns. Hierarchical clustering identified cytokine groups having similar expression profiles across subphenotypes and in patients having similar expression profiles within each subphenotype (Fig. 1). Some cytokines presented large variations in their expression levels. Two clusters of cytokines were identified. The first consisted of cytokines abundantly present in patient plasma—GM-CSF, Eotaxin, IL-1α, MCP-1, IL-10, MIP-1β, MIP-1α, IL-8, IL-6, TNF-α, IFN-α2, IL-7, G-CSF, and IP-10 (Additional file 2: Figure S2); the second, characterized by lower cytokine levels, comprised IL-3, IL-5, IL-15, TNF-β, IL-2, IL-13, IL-4, IL-12p40, IL-17, IFN-γ, IL-12p70, and IL1-β (Additional file 3: Figure S3). All malarial subgroups, but not controls (EC, SEPT, and ENC), exhibited increased levels of TNF-β, IL-12p70, and IL-4. However, IL-15 and IL-12p40 levels were higher in SEPT and ENC patients. All malarial subgroups showed large variation in cytokine expression and had subpopulations with very low cytokine levels. Medians of cytokine levels tended to increase with disease severity.

**Fig. 2:**
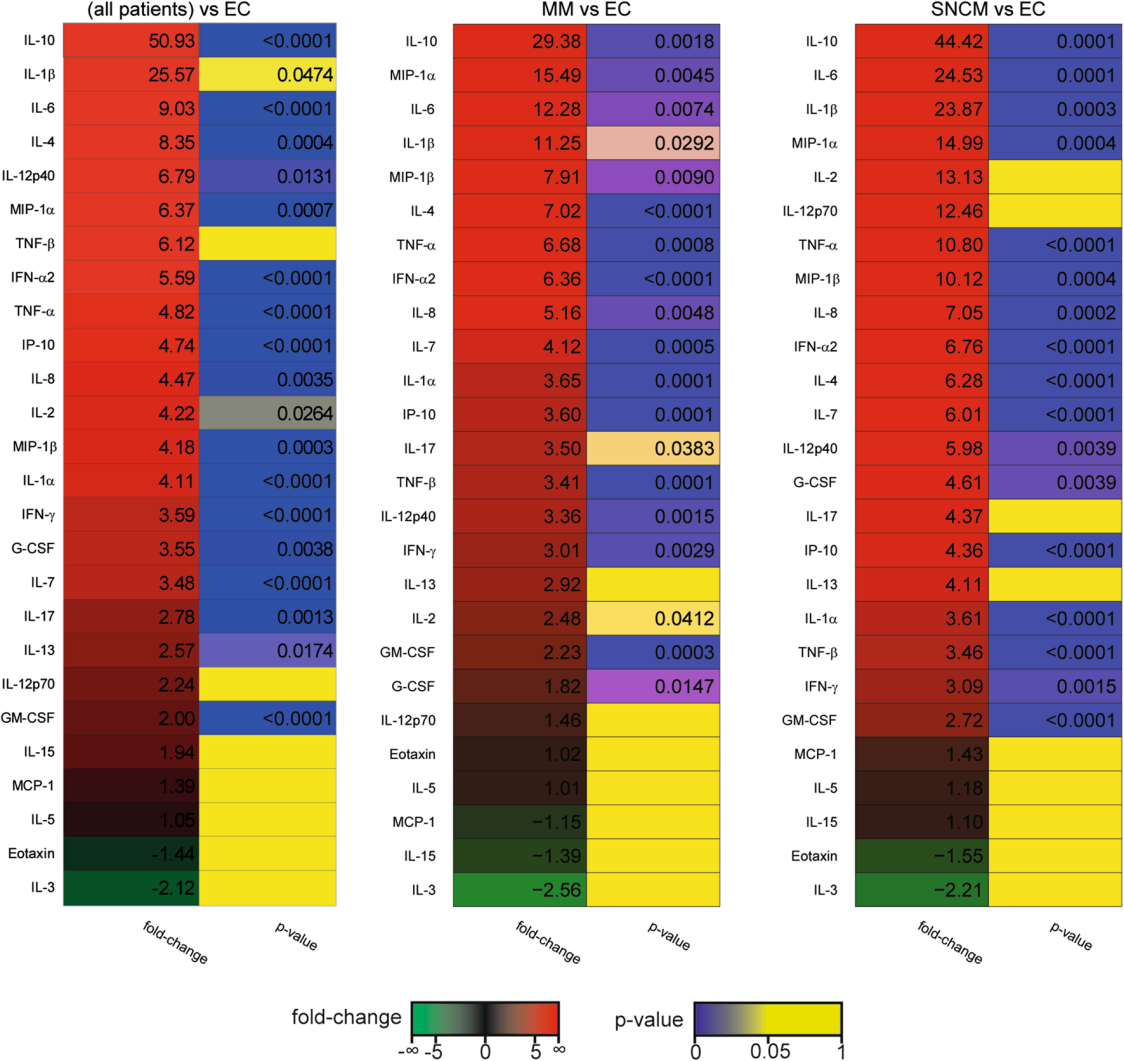
Heatmaps of observed fold-changes in cytokine concentrations in malaria groups as compared to endemic controls. *Each panel* shows a heatmap of the observed fold-change in cytokine concentration in the plasma for all patients and for particular subgroups of malarial patients with respect to the indicated control group. Heatmaps were sorted by fold-change values. Associated p values obtained using the Student’s *t* test are also indicated

**Fig. 3:**
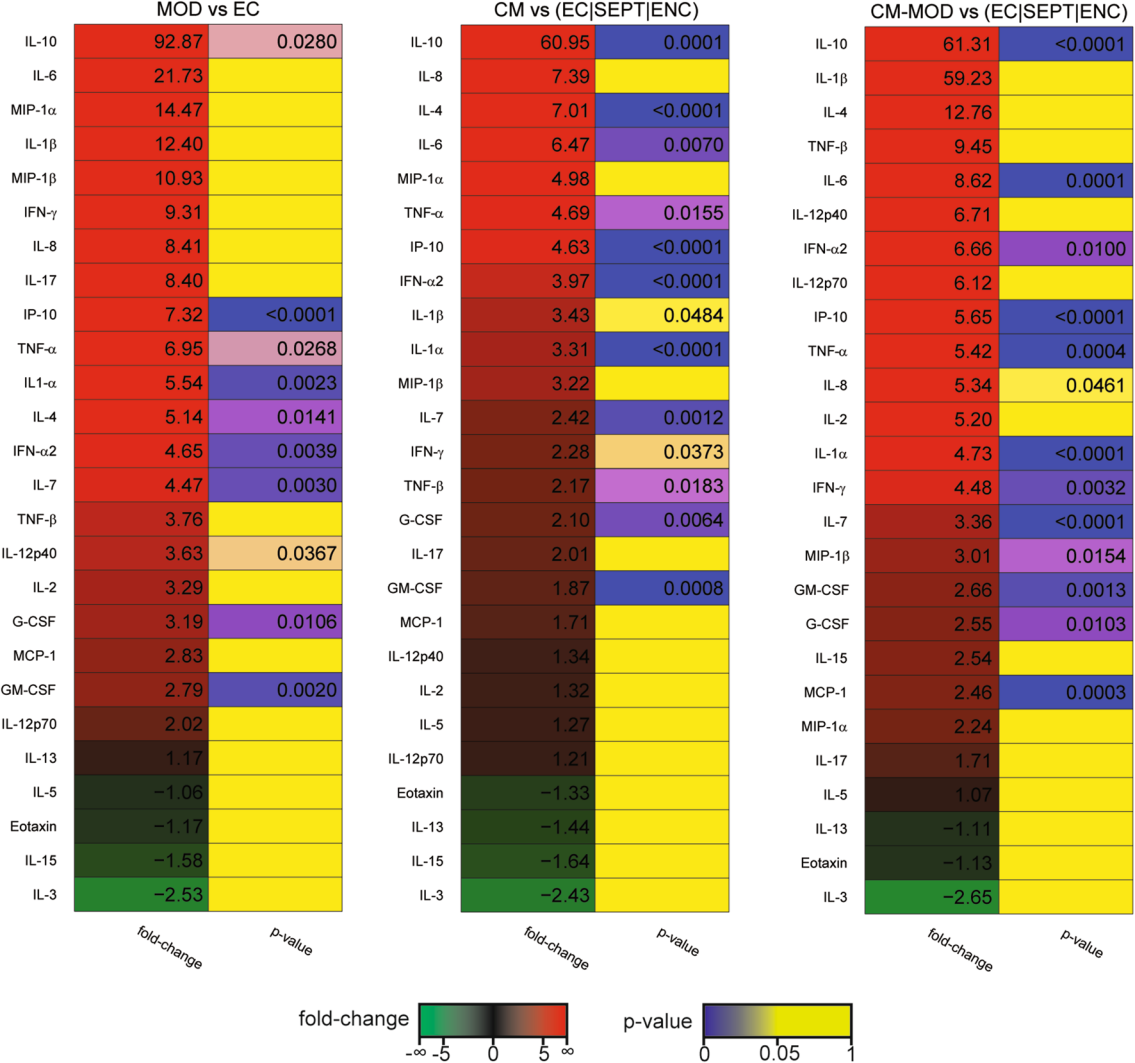
Heatmaps of observed fold-changes in cytokine concentrations in severe malaria groups as compared to sepsis and encephalitis. *Each panel* shows a heatmap of the observed fold-change in cytokine concentration in the plasma for all patients and for particular subgroups of malarial patients with respect to the indicated control group. Heatmaps were sorted by fold-change values. Associated p values obtained using the Student’s *t* test are also indicated

We next aimed to identify the cytokines differentially expressed between the different biological conditions. First, we compared all patients with EC and each malaria subgroup with EC using the Student’s *t* test. Additionally, we compared the CM and CM-MOD groups with a group comprising SEPT and ENC—non-parasitic diseases with brain/multi-organ involvement—and EC. Of 26 cytokines/chemokines, 19 increased significantly during malaria. When cytokine profiles of patients in individual categories were compared to the cytokine profiles of controls, distinct patterns emerged. Many more cytokines were upregulated in MM and SNCM patients than in MOD, CM, and CM-MOD patients (Fig. 2). IL-10 clearly rose with severity (~fold increase of 29, 44, 93, and 61 in MM, SNCM, MOD, and CM/CM-MOD, respectively; Fig. 3).

Of note, there was no correlation between age, sex, and disease phenotype or cytokine levels in all the groups studied. In agreement with studies previously reported in Odisha, a higher percentage of males were observed among all malaria groups [31].

### Cytokines/chemokine clusters differentiating MM from CM in Gondia do not differentiate them in Odisha

**Fig. 4:**
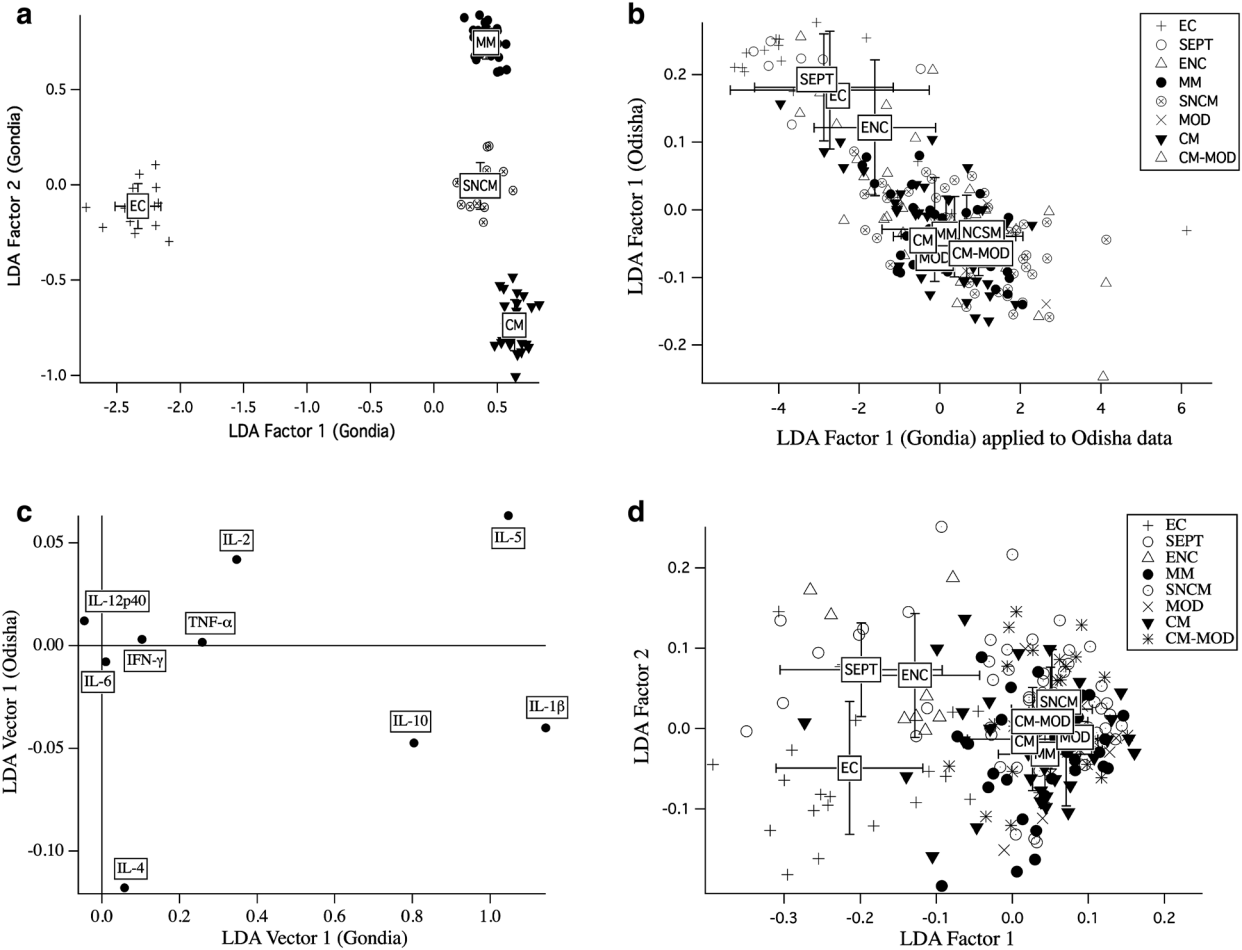
Linear discriminant analysis. **a** Discrimination of malaria clinical subphenotypes in Gondia patients. Factor 1 discriminates infection; factor 2 discriminates different manifestations. **b** Projection of Gondia LDA factor 1 using data from Odisha, compared to direct LDA discrimination of Odisha subjects by the same cytokines studied in Gondia: both 1st factors discriminate *P. falciparum* infection. **c** Factor loads of the respective first LDA factors. **d** LDA discrimination of the patient subgroups studied in Odisha using all 26 cytokines

We investigated whether the cytokine clusters that discriminated MM from CM in Gondia [23] could differentiate these subgroups in Odisha. We selected cytokines common to both studies, i.e., IFN-γ, IL-1β, IL-2, IL-4, IL-5, IL-6, IL-10, IL-12p40, and TNF-α, and performed an LDA. Resulting Gondia LDA factors discriminated infection (Gondia LDA factor 1) and clinical manifestations (Gondia LDA factor 2; Fig. 4a). We projected the Odisha data onto Gondia LDA factor 1 and calculated a new LDA factor for the Odisha dataset (Fig. 4b). Although these factors distinguished malaria patients from controls (Fig. 4b), the cytokines that distinguished malarial subgroups in Odisha were very different from those in Gondia (Fig. 4c). Furthermore, the clear separation of clinical subgroups observed in Gondia (factor 2) was not seen in Odisha. However, LDA of the entire dataset distinguished the control groups from malaria subgroups and MOD from CM-MOD (Fig. 4d).

### Identification of cytokines that differentiate Odisha MOD, CM, and CM-MOD subphenotypes

**Fig. 5:**
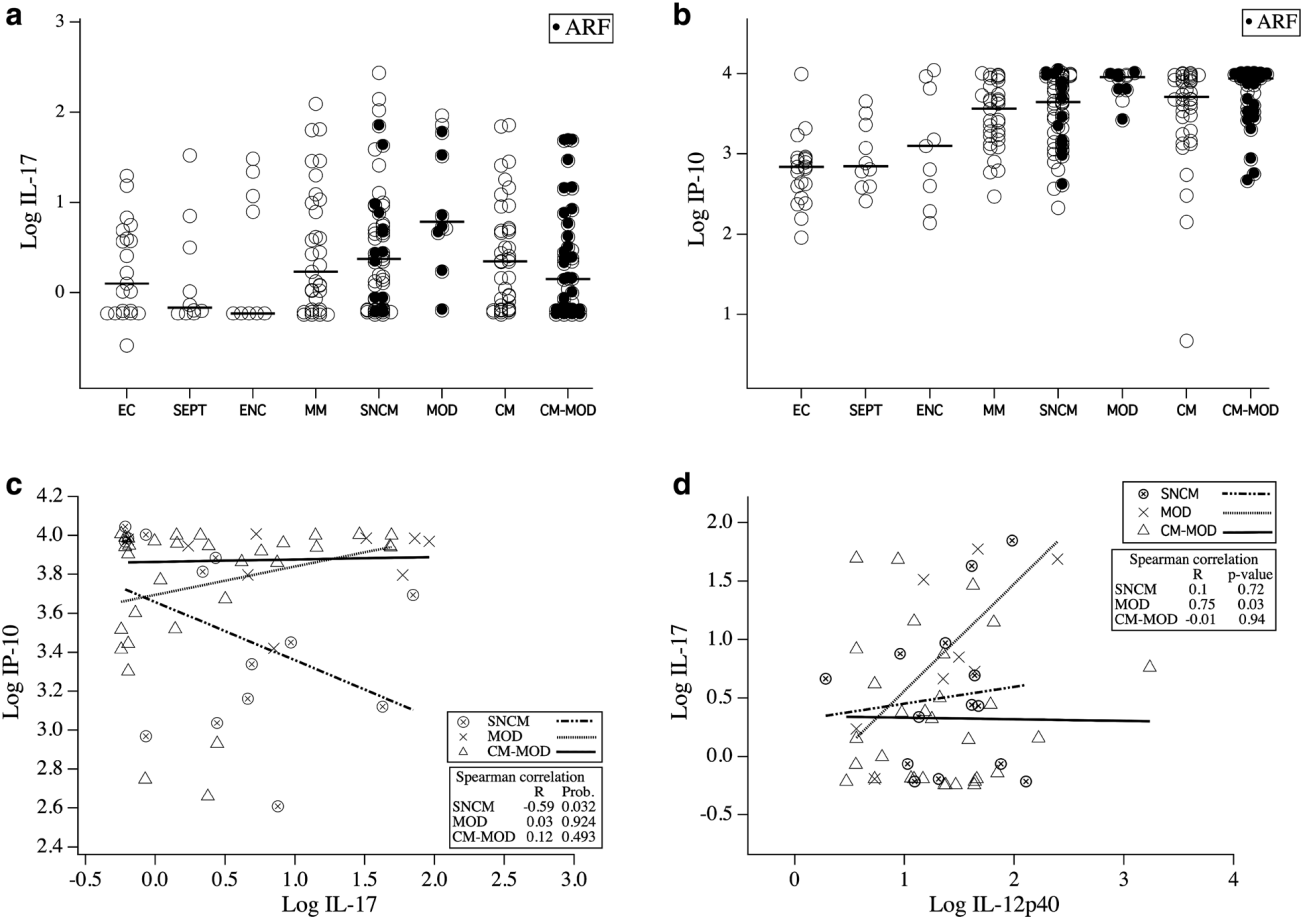
Cytokine levels in different malarial subgroups. Levels of log_10_-transformed (ng/mL of plasma) IL-17 (**a**) and IP-10 (**b**) were measured and plotted according to groups. *Horizontal bars* indicate the median. *Black dots* represent patients with ARF (**a** and **b**). Correlations between these two cytokines for SNCM, MOD, and CM-MOD patients with ARF are represented in (**c**). Data were analyzed using the Spearman correlation test. p values ≤0.05 were considered as significant. **d**. Relationship between levels of IL-17 and IL-12p40 among patients developing ARF.Levels of IL-17 and IL-12p40 were plotted for ARF patients from SNCM, MOD and CM-MOD groups. Data were analyzed using the Spearman correlation test. p ≤ 0.05 was considered as significant

Using the Student’s *t* test, we characterized cytokine patterns associated with each severe malaria subgroup and patterns differentiating MM from severe malaria (SNCM, MOD, CM, CM-MOD). The MOD and CM-MOD groups were statistically indistinguishable (Additional file 4: Figure S4). Only IL-1α levels were significantly different between the CM-MOD and CM groups, with a fold-change of 1.43 (p = 0.009). By contrast, IP-10 and GM-CSF distinguished CM from the MOD group, with fold-changes of −1.46 (p = 0.006) and −1.66 (p = 0.03), respectively. We found the cytokine set associated with each severe malaria subgroup using logistic regression. This allowed determination of the discriminant power of some cytokines, accounting for multivariate dependence between predictors. Once a cytokine was identified as discriminant, we characterized its effect through an odds-ratio (OR). When the severe non-cerebral (SNCM + MOD) group was compared to the cerebral malaria (CM + CM-MOD) group (Additional file 5: Table S1), only MIP-1α discriminated the two (OR = 0.71; p = 0.0003. Within the SNCM + MOD group, only IL-17 (OR = 0.63; p = 0.04) and IP-10 (OR = 0.08; p = 0.03) differentiated SNCM from MOD. Levels of both cytokines were higher in MOD than SNCM. Most patients (SNCM, MOD, and CM-MOD) with ARF exhibited high IL-17 (Fig. 5a) or IP**-**10 (Fig. 5b) levels. In addition, in SNCM patients with ARF, IP-10 concentrations were negatively correlated to IL-17 (R = −0.59; p = 0.03) (Fig. 5c). Interestingly, levels of IL-17 and IL-12p40 were also correlated in ARF patients from the MOD group (Fig. 5d).

**Fig. 6:**
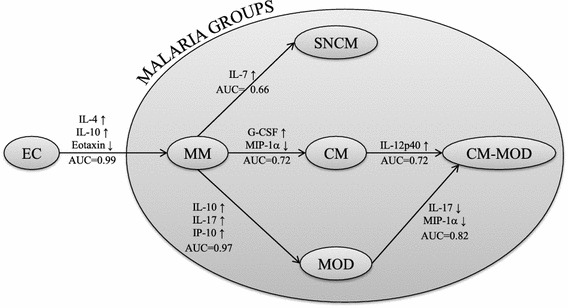
Logistic regression. Schematic representation of the results of the logistic regressions applied to discriminate patient subphenotypes. Each regression is represented by a *long arrow* between the two subgroups under consideration (from reference subgroup to target subgroup). On each *arrow* is inscribed: the area under the receiver operating characteristic curve (AUC), indicating the quality of the discrimination; the significant cytokines and the sign of their effect (↑ if an increase of the value of the cytokine is in favor of the target subgroup; ↓ if an decrease of the value of the cytokine is in favor of the target subgroup)

Cytokine patterns in severe malaria subgroups were then compared to identify cytokines contributing to a given clinical subphenotype (Additional file 5: Table S1). Levels of GM-CSF and IL-17 were significantly higher in the MOD than in the CM group, allowing for their differentiation (OR = 0.2; p = 0.03 and OR = 0.5; p = 0.02 for GM-CSF and IL-17, respectively). Besides IL-17, high levels of MIP-1α also differentiated CM-MOD from MOD. This approach was further used to define cytokines that discriminated all clinical malaria subgroups. The results are represented in Fig. 6. This cytokine/chemokine network could be helpful in stratifying severe malaria manifestations into clinical sub phenotypes (Additional file 6: Table S2).

## Discussion

Falciparum malaria, whose manifestations vary from mild clinical illness to coma, severe anemia, respiratory distress, shock, and MOD, is characterized by marked changes in cytokine production. Therefore, we examined cytokine patterns in a well-characterized cohort of malaria patients in Odisha, India, and compared them to healthy, uninfected controls to determine whether differences in plasma cytokine levels could characterize severe malaria subsets. In Odisha, severe falciparum malaria is often associated with ARF, and over 86 % deaths are due to CM-associated renal failure [5]. In this study too, 88 % of MOD and 100 % of CM-MOD patients had ARF. Reasons for the increase in ARF, mechanisms precipitating it, and the relationship between cytokines and ARF remain unclear.

We could clearly distinguish malaria patients from healthy controls and from patients of the severe infections encephalitis and sepsis. However, the cluster of cytokines—TGF-β, TNF-α, IL-10, and IL-1β—that differentiated CM from other falciparum malaria manifestations in Gondia could not distinguish severe malaria cases in Odisha [23]. Indeed, in this population, cytokine responses differed from published reports. As in other studies, IL-1β was the only cytokine that contributed significantly to the discrimination of CM in Gondia [23]. Surprisingly, in Odisha patients, IL-1β levels decreased with disease severity and were highest in severe malaria patients with no brain injury. Moreover, TNF-α, linked to disease severity and death in several studies, was not thus linked in this population [7, 12]. These differences could be attributed to multiple factors. First is the difference in malaria endemicity. Gondia has an API of <1 %, whereas coastal Odisha has an API of >5 % [24]. The sites also differ in range of manifestations—MOD, observed in Odisha, was absent in Gondia. Factors such as co-infections, population genetics, and environment could also influence response. Our data suggest that the pathophysiology of malaria, cytokine profiles, and range of manifestations may vary greatly across regions; this must be taken into consideration when designing studies and interpreting results.

To detect cytokine clusters distinguishing severe malaria subphenotypes in Odisha, we used an ensemble of methods including the capacity to predict the outcome using a test data set. The cytokine expression heatmaps did not reveal distinctive patterns associated with particular subphenotypes, IL-17 and MCP-1 levels increased during MOD and IL-1β during SNCM. Our results are consistent with previous studies implicating dysregulation of pro- and anti-inflammatory cytokines/chemokines in disease progression [32–34].

All malarial patients showed higher IL-4 and IL-10 levels and lower Eotaxin levels than EC. It is noteworthy that cytokine identification in our logistic regression model is not subject to confounding effect. For example, Eotaxin alone has a marginal effect when EC are compared to malaria patients, whereas it becomes significant when calculated with covariates (IL-4 and IL-10). However, the decrease in Eotaxin levels was not unique to malaria; a similar decline was observed in SEPT and ENC.

Mild disease was distinguished from cerebral outcomes (CM + CM-MOD) by high levels of MCP-1 associated with low levels of MIP-1α. MCP-1 is thought to contribute to increased permeability of the blood–brain barrier [35], and high levels of MCP-1 have been reported in patients with several neuroinflammatory diseases, including multiple sclerosis, cerebral ischaemia, and HIV-1 encephalitis [36, 37]. To our knowledge, this is the first study to show increased MCP-1 levels in CM patients.

IP-10, a potent pro-inflammatory chemokine, has been implicated in the recruitment and migration of leukocytes to the brain in experimental CM [38, 39]. High levels of IP-10 in CSF and plasma are proposed predictors of severe and fatal CM in Indian populations [40]. In our study, IP-10 levels correlated significantly with malarial severity and were highest in MOD patients. Median IP-10 levels were similar in CM and SNCM patients. Nevertheless, 70 % of the CM-MOD group had very high IP-10 concentrations; in 50 %, the disease proved fatal. This suggests that IP-10 alone may not be a predictor of fatal CM in the Odisha population.

A key study finding was that IL-17 was higher in MOD than in other severe malaria subgroups, including CM and CM-MOD. IL-17 and the Th17 cells that produce it are involved in the development of inflammatory renal diseases, such as lupus nephritis and nephrotoxic nephritis, as well as in allograft rejection [41]. Our observations suggest a role for the IL-17/Th17 pathway in renal inflammatory pathology in falciparum infections in Odisha. Thus, an excess of IL-17 may promote MOD, particularly ARF. Notably, most CM-MOD mortalities with ARF exhibited the highest IL-17 plasma concentrations. IL-12p40 was the only cytokine that segregated CM from CM-MOD. IL-12p40 is a shared subunit between IL-12 and IL-23 [42]. Interestingly, levels of IL-17 and IL-12p40 are correlated in ARF patient from the MOD group. As IL-12p40, but not IL-12p70, distinguishes CM and CM-MOD, our results may suggest a critical role for IL-23 in MOD. This hypothesis is based on the fact that IL-23 promotes the differentiation of Th17 cells, and contributes also to renal tissue injury [43]. Elucidating the pathways that regulate Th17 differentiation, Th17 effector functions, and IL-17 secretion during malaria may clarify the role of inflammatory processes in ARF etiology.

IL-17 associated with high GM-CSF distinguished MOD from CM group and was segregated from the CM-MOD group when associated with high levels of MIP-1α. GM-CSF promotes neutrophil recruitment and function, and it is implicated in neuroinflammation [44]. Distinct and counter-regulatory pathways have been associated with the generation of IL-17 and GM-CSF producing cells [45]. It is tempting to speculate that high levels of IL-17 may protect from malaria-associated neurological complications although beyond a threshold it may result in renal complications.

## Conclusion

We report distinct differences in cytokine production correlating with infection and disease severity in Odisha populations. We also report discrete cytokine patterns in subphenotypes of severe malaria. Limitations of examining cytokine production at single time points and in circulation (rather than local microenvironments) complicate the interpretation of these results. Nevertheless, the differences in cytokine patterns of MM and severe malaria patients as well as the distinct cytokine profiles of CM, CM-MOD, and MOD patients may prove insightful. Therefore, identification of biomarkers that are sensitive and specific to disease severity may serve as prediction scoring system to prognosticate disease outcome in adult patients. A broader understanding of the interplay of cytokines/chemokines produced in response to falciparum infection in different populations and their relation to disease severity may assist in developing more targeted interventions.

## Additional files

10.1186/s12967-015-0731-6 Multidimensional scaling representation of samples on the basis of their cytokine profiles. Each dot represents a cytokine profile of a sample plotted in the intensity space of the expression signals. Pairwise distances between dots are proportional to the Euclidean distances between samples. Euclidean distances have been calculated based on the log10 transformed signals and using all measured cytokines values. Convex hulls (i.e. the smallest convex set containing the points) delineate each biological condition. The Kruskal Stress criterion shown in the representation quantifies the quality of the representation as a fraction of the information lost during the dimensionality reduction procedure.
10.1186/s12967-015-0731-6 Set of cytokines abundantly produced during malaria. Boxplots and plot-jittering representations showing cytokine distributions across different subpehnotypes of malarial patients and controls. Box plots show the first and third quartiles, together with the medians of the cytokines levels expressed as log10-transformed values (ng/mL of plasma) across categories of malarial patients (MM, SNCM, MOD, CM, CM-MOD) and controls (EC, SEPT, ENC). Individual cytokines within each category are represented using a jittering method in order to reduce display overlap.
10.1186/s12967-015-0731-6 Set of cytokines characterized by relatively lower levels of expression during malaria. Boxplots and plot-jittering representations showing cytokine distributions across different subpehnotypes of malarial patients and controls. Box plots show the first and third quartiles, together with the medians of the cytokines levels expressed as log10-transformed values (ng/mL of plasma) across categories of malarial patients (MM, SNCM, MOD, CM, CM-MOD) and controls (EC, SEPT, ENC). Individual cytokines within each category are represented using a jittering method in order to reduce display overlap.
10.1186/s12967-015-0731-6 Heatmaps of cytokine signatures between MOD, CM, and CM-MOD groups. Heatmaps show the cytokine fold-change with the associated p-values for comparison between CM and MOD, CM-MOD and MOD, and CM-MOD and CM groups. Each heatmap has been sorted by the fold-change values. Fold-change values and p-values are indicated for all cytokines.
10.1186/s12967-015-0731-6 Discriminant cytokines distinguishing controls and malaria groups.
10.1186/s12967-015-0731-6 Univariate significance of cytokines that differentiates malarial sub phenotypes.

## Abbreviations

CM: cerebral malaria
MOD: multiple organ dysfunction
CM-MOD: cerebral malaria with MOD
ARF: acute renal failure
MM: uncomplicated malaria
SNCM: severe non cerebral malaria
EC: asymptomatic carriers
SEPT: sepsis
ENC: encephalitis
MDS: multidimensional scaling
SVD: single value decomposition
