# Supplementary figures and images for: Evidence of IL-17, IP-10, and IL-10 involvement in multiple-organ dysfunction and IL-17 pathway in acute renal failure associated to Plasmodium falciparum malaria
Source: J Transl Med. 2015 Nov 24;13:369. doi: 10.1186/s12967-015-0731-6 (PMC4658812; doi:10.1186/s12967-015-0731-6)

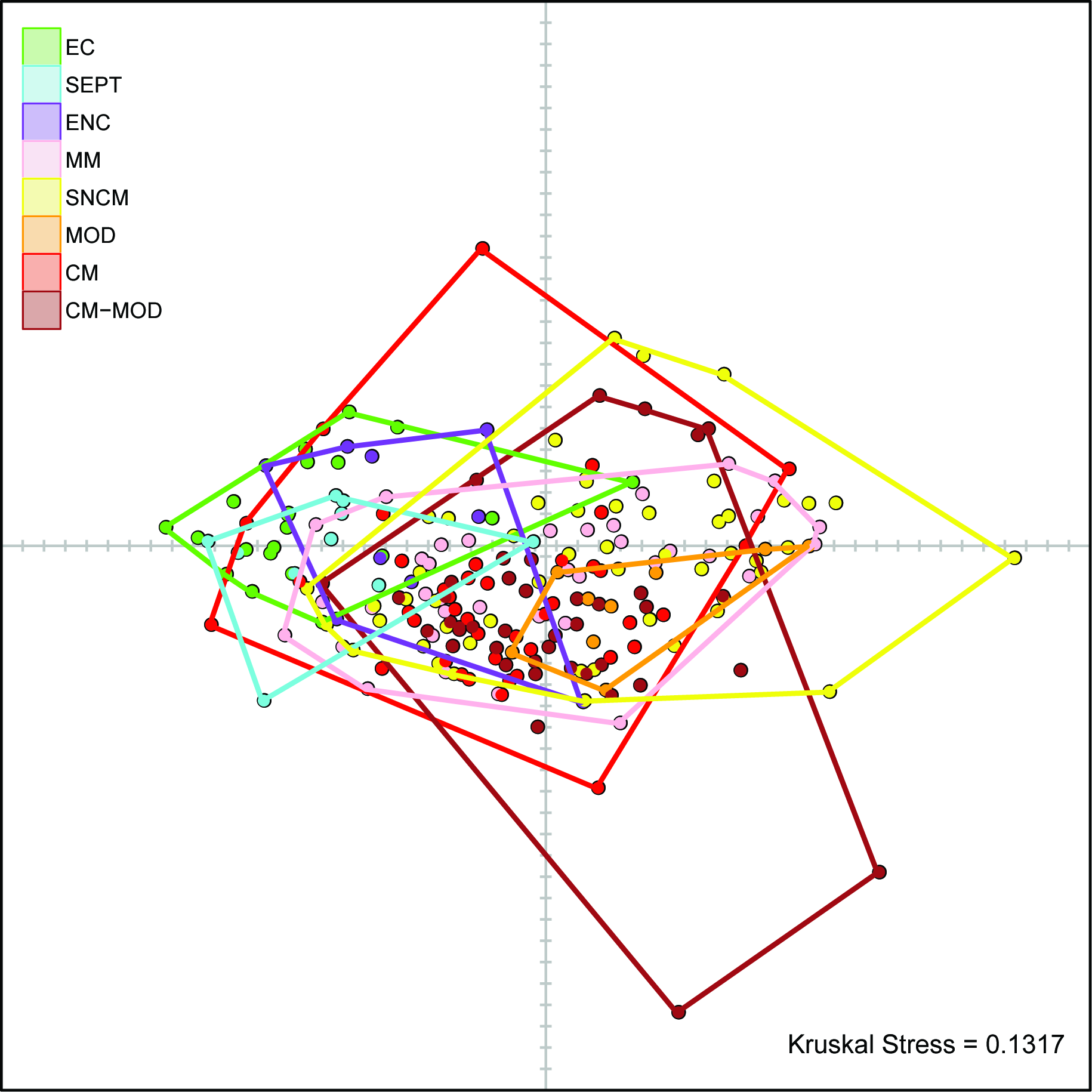

Supplement: Supplementary file 1 — 10.1186/s12967-015-0731-6 Multidimensional scaling representation of samples on the basis of their cytokine profiles. Each dot represents a cytokine profile of a sample plotted in the intensity space of the expression signals. Pairwise distances between dots are proportional to the Euclidean distances between samples. Euclidean distances have been calculated based on the log10 transformed signals and using all measured cytokines values. Convex hulls (i.e. the smallest convex set containing the points) delineate each biological condition. The Kruskal Stress criterion shown in the representation quantifies the quality of the representation as a fraction of the information lost during the dimensionality reduction procedure. [file 12967_2015_731_MOESM1_ESM.tiff]

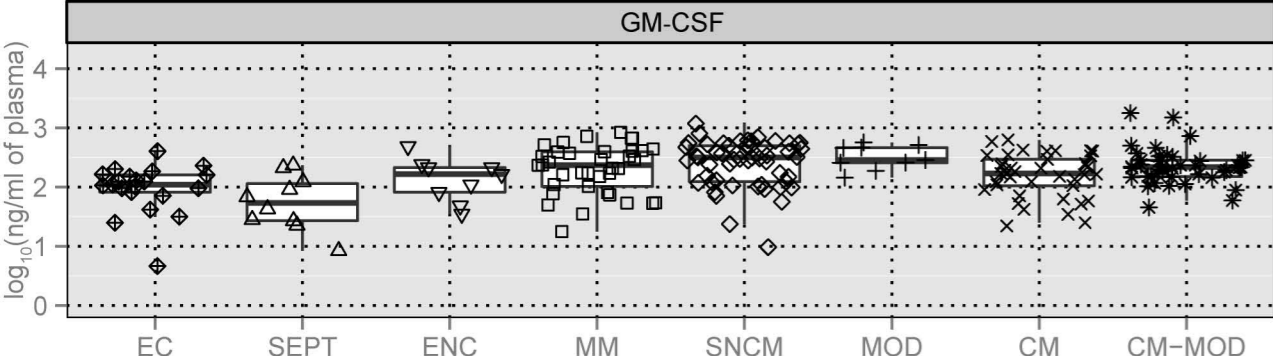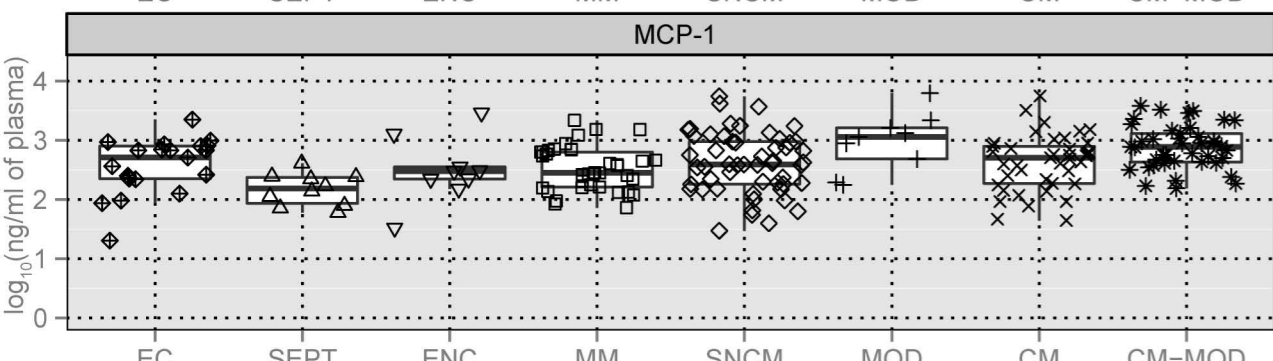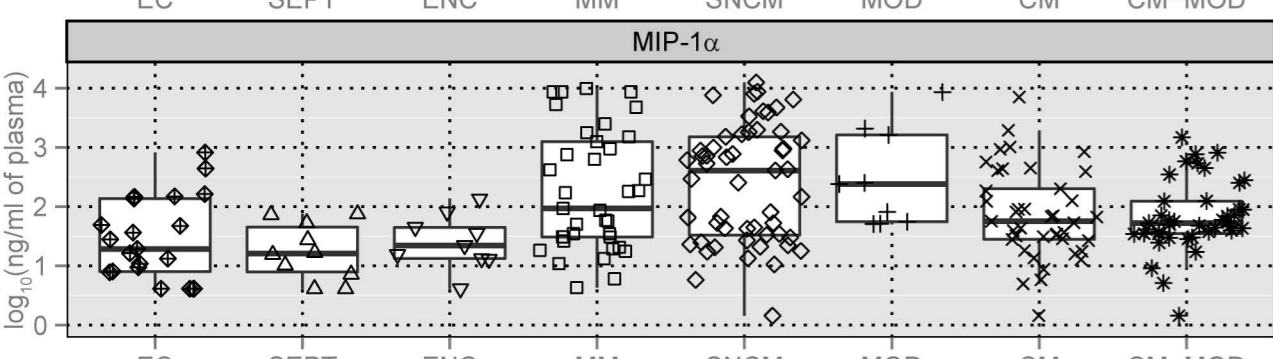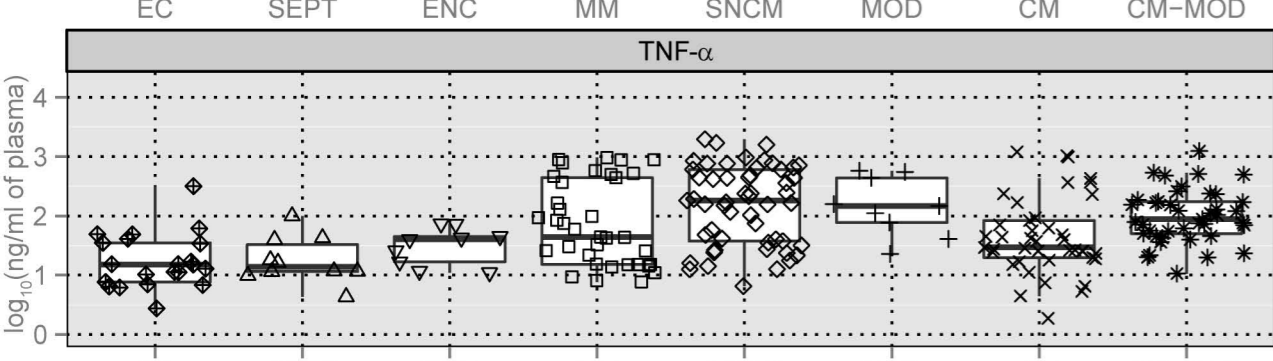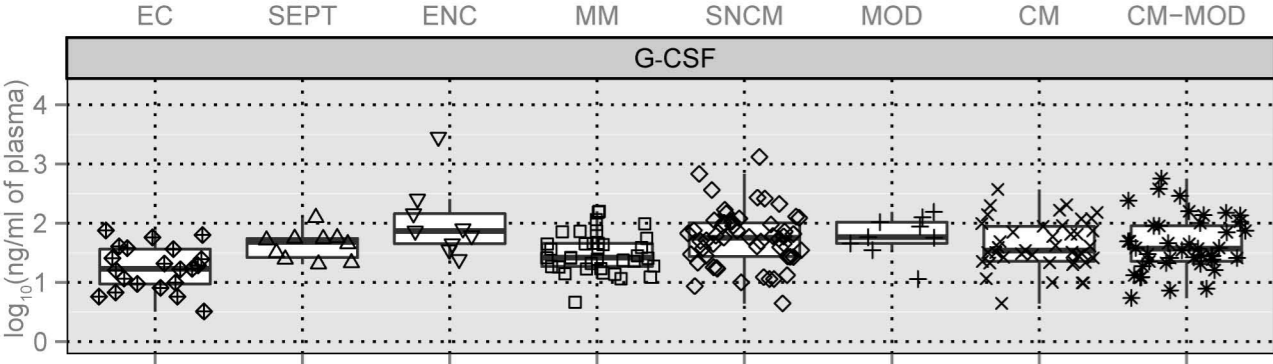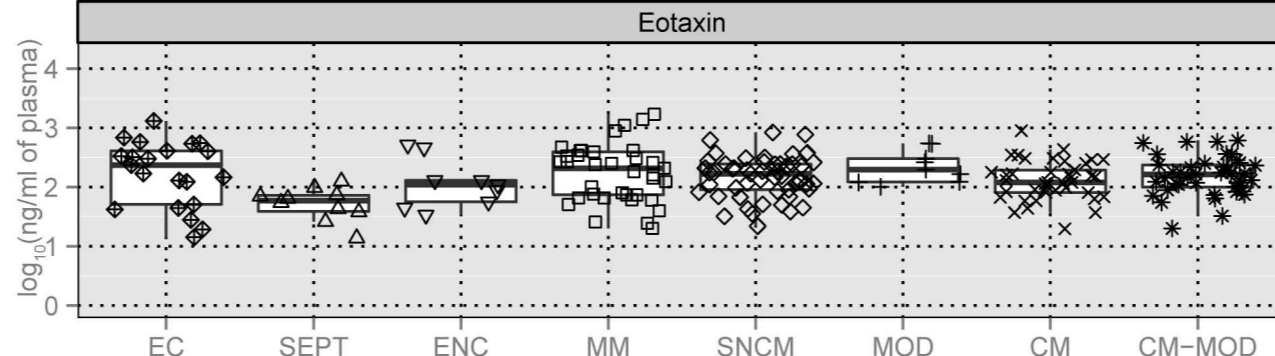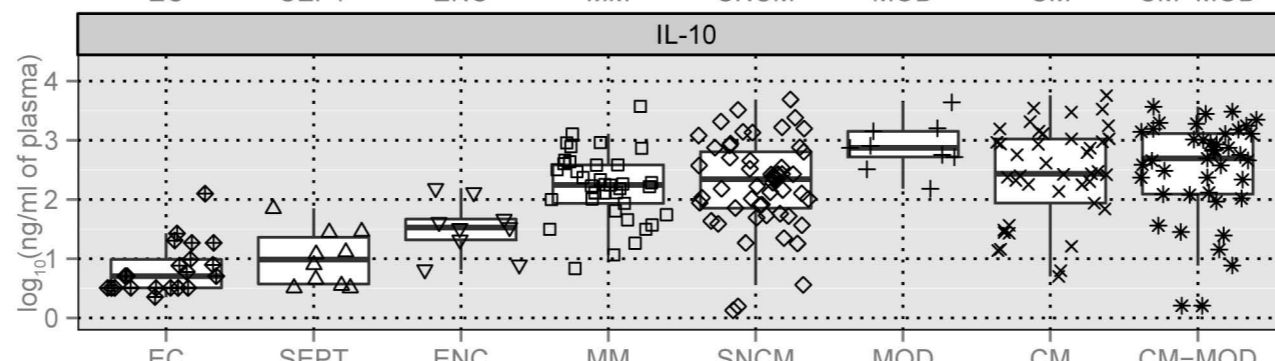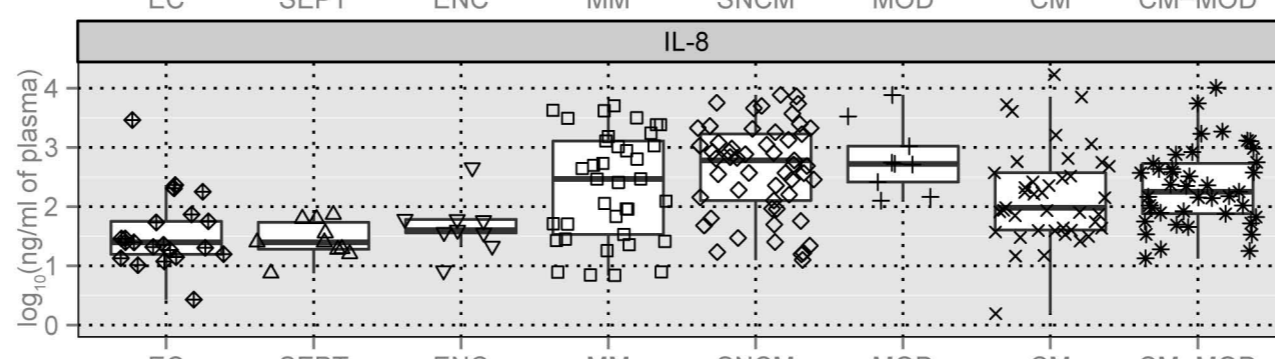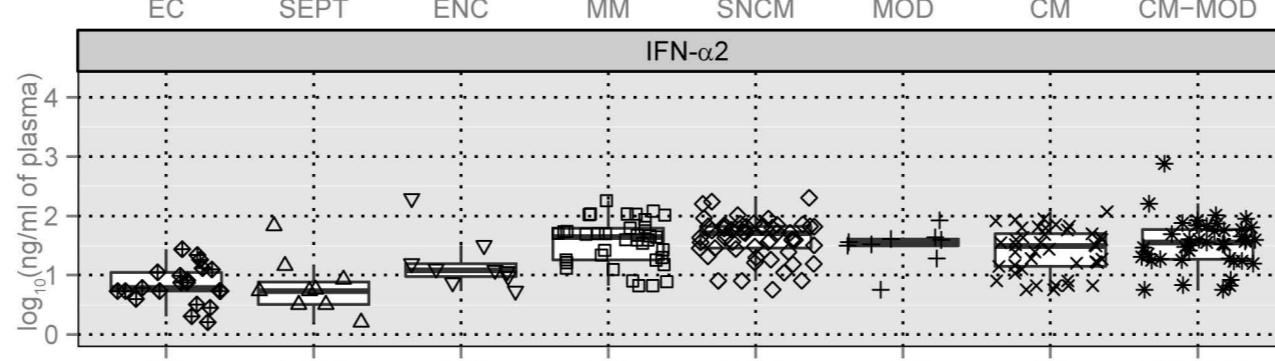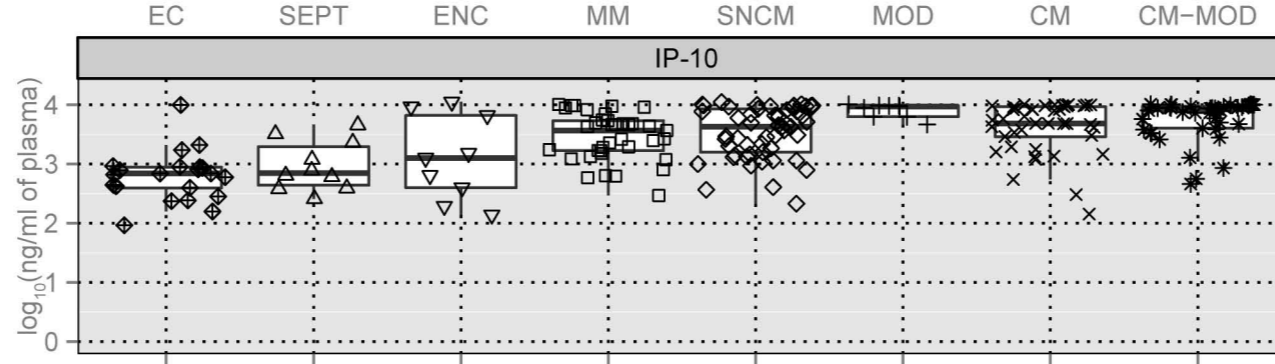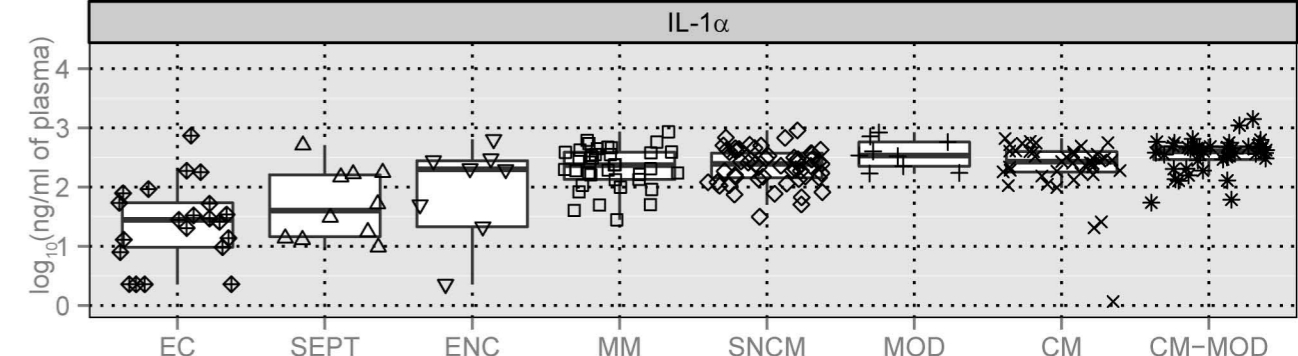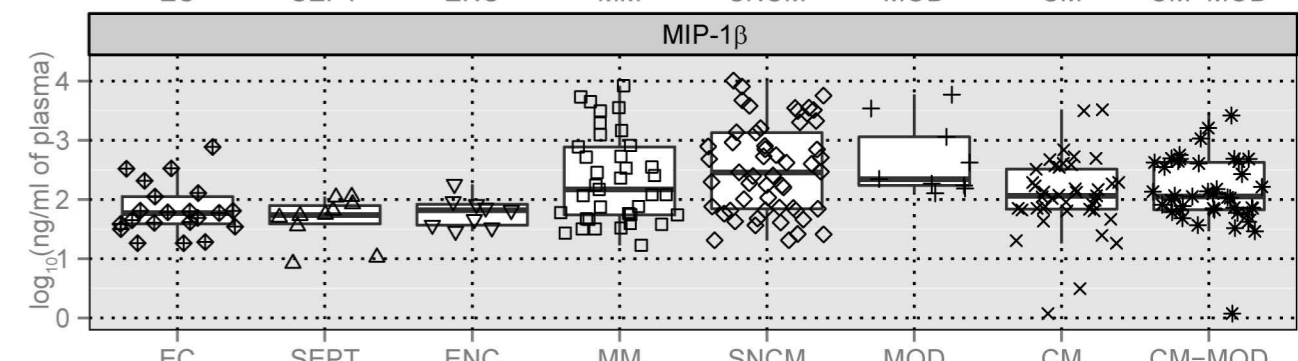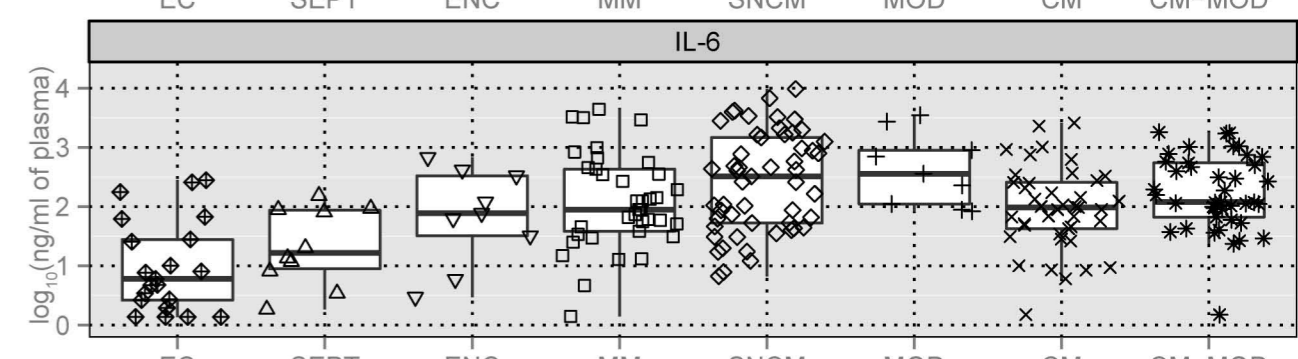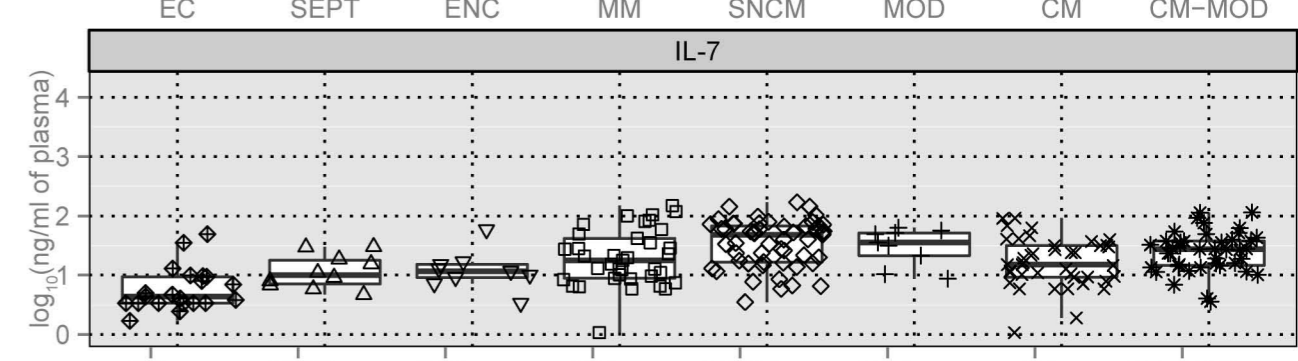

Supplement: Supplementary file 2 — 10.1186/s12967-015-0731-6 Set of cytokines abundantly produced during malaria. Boxplots and plot-jittering representations showing cytokine distributions across different subpehnotypes of malarial patients and controls. Box plots show the first and third quartiles, together with the medians of the cytokines levels expressed as log10-transformed values (ng/mL of plasma) across categories of malarial patients (MM, SNCM, MOD, CM, CM-MOD) and controls (EC, SEPT, ENC). Individual cytokines within each category are represented using a jittering method in order to reduce display overlap. [file 12967_2015_731_MOESM2_ESM.pdf]

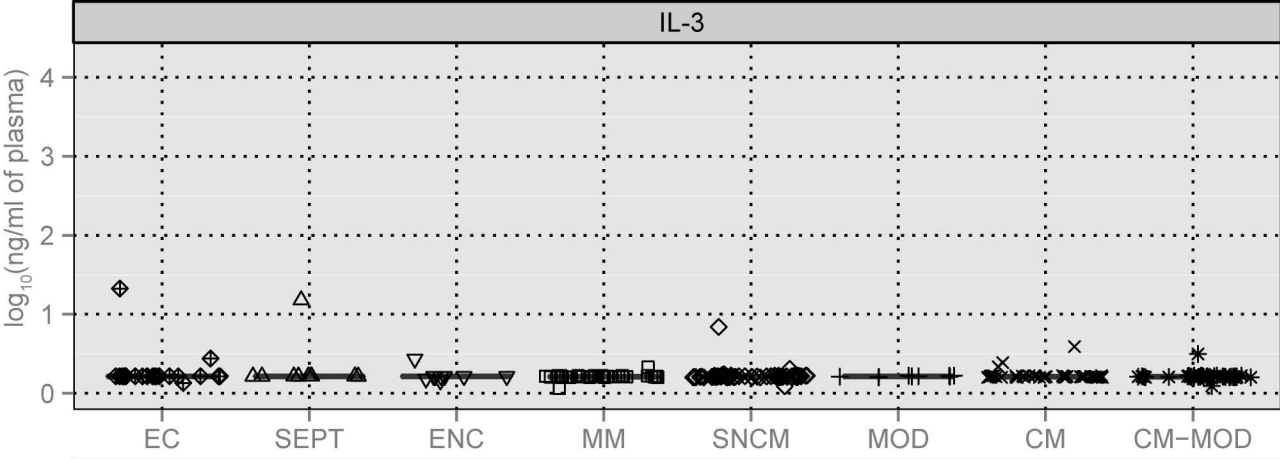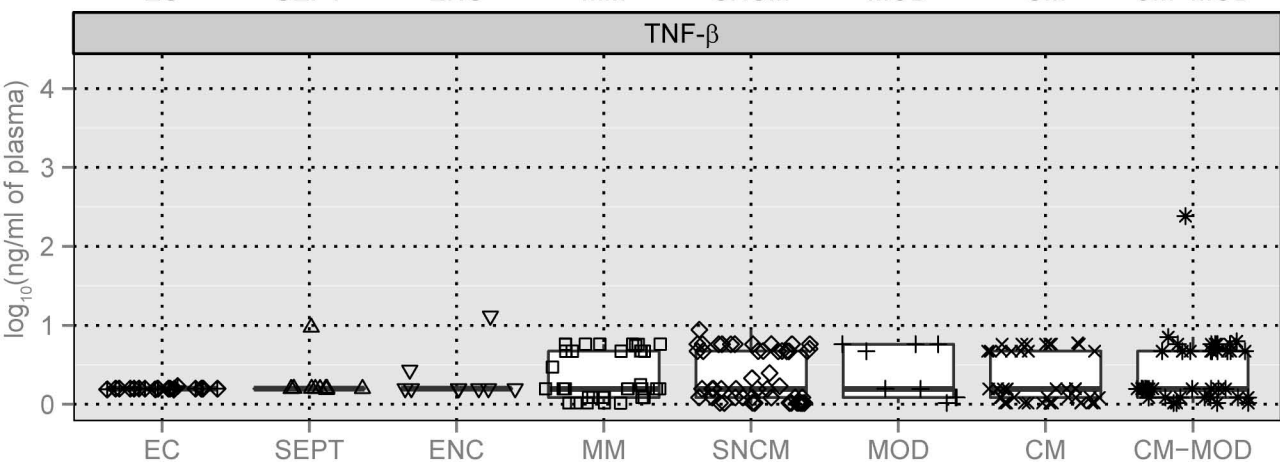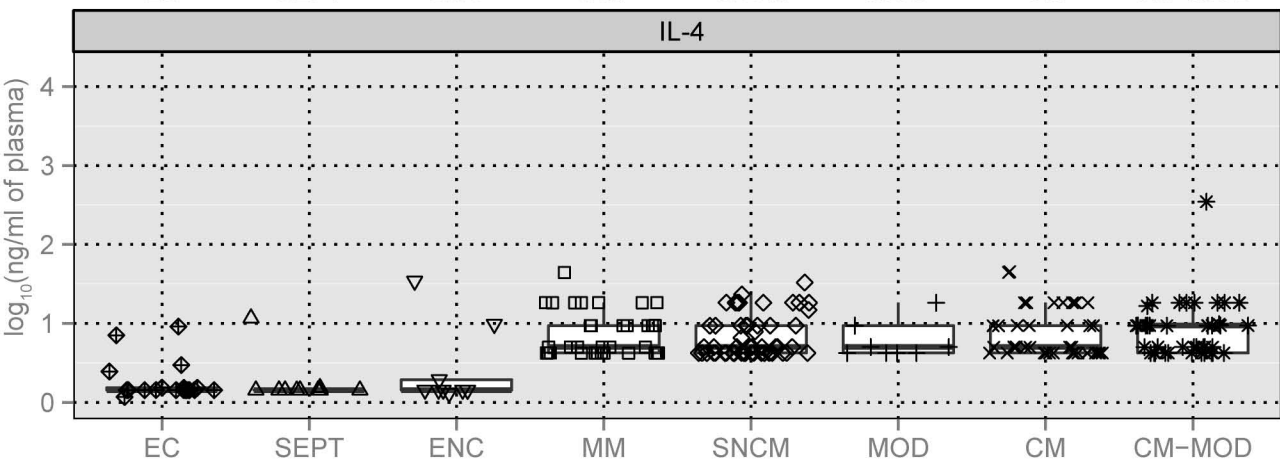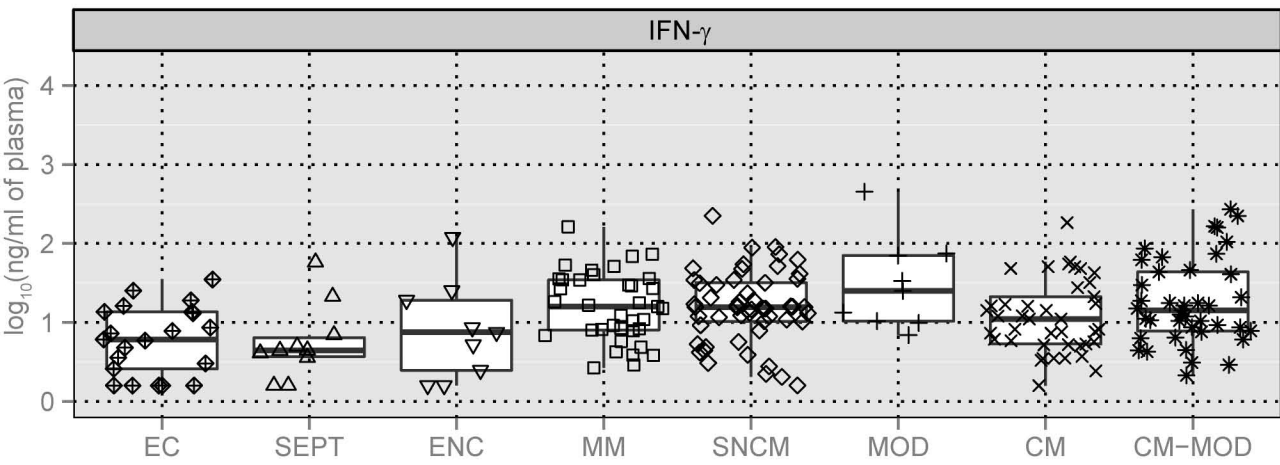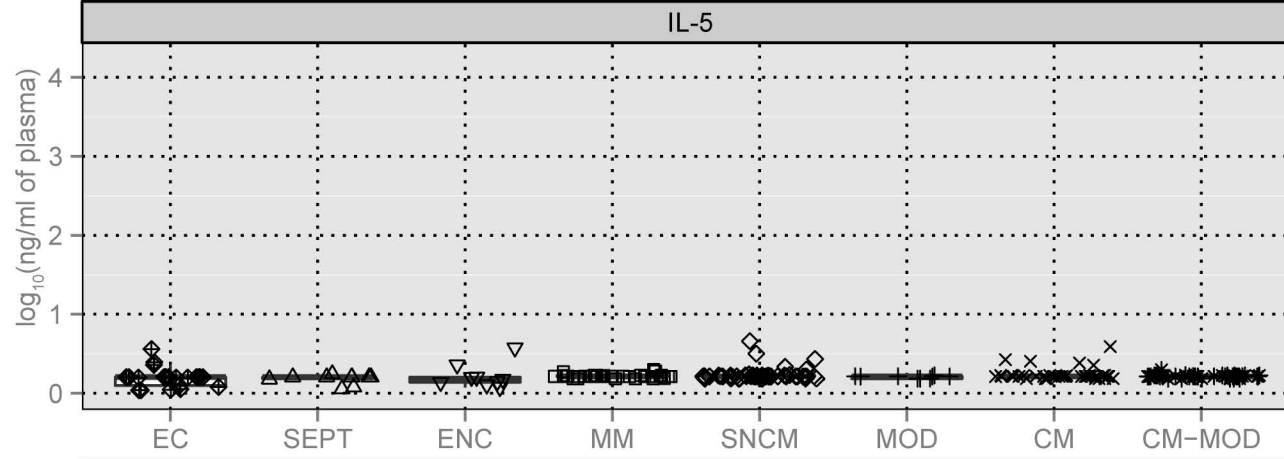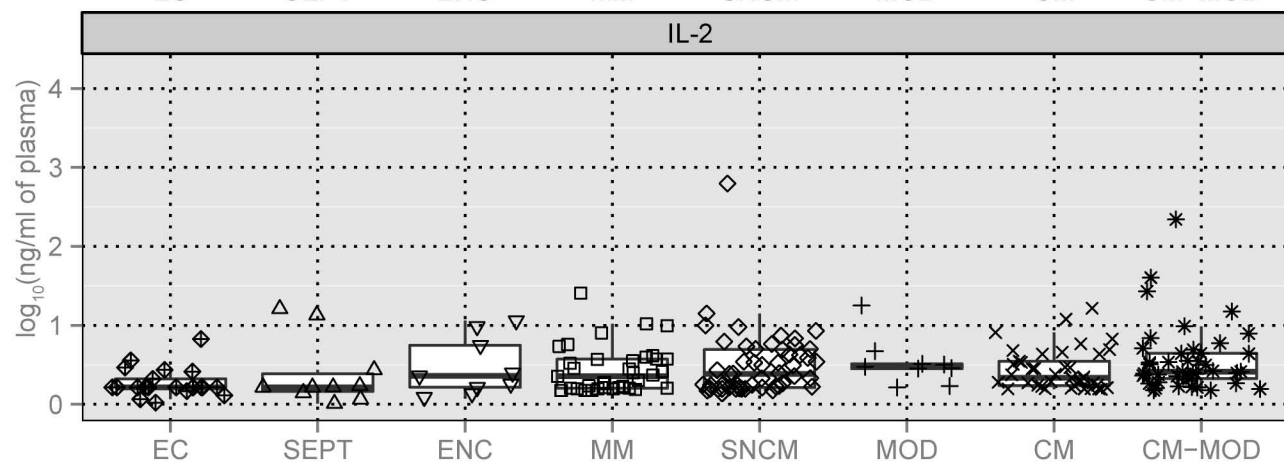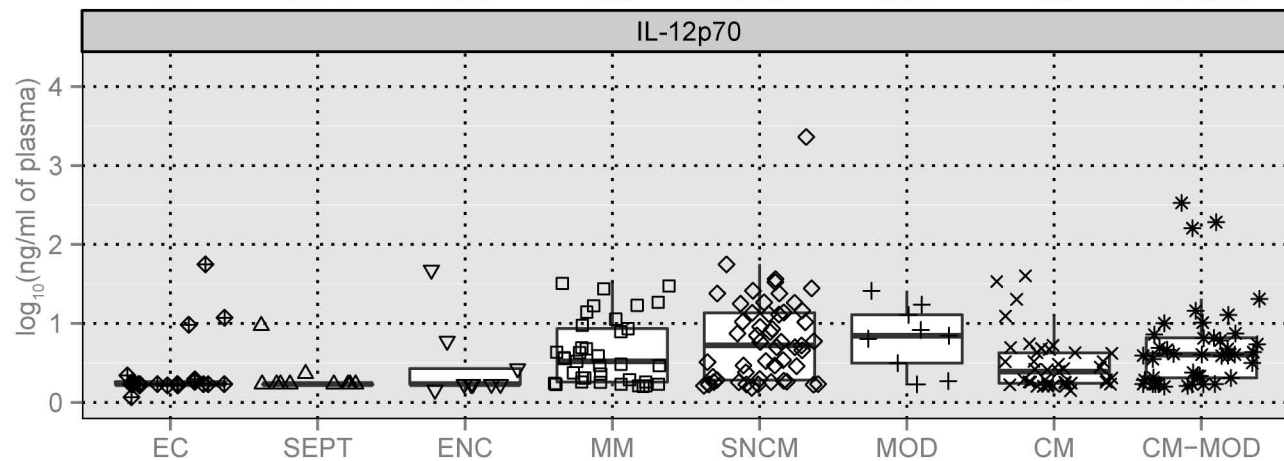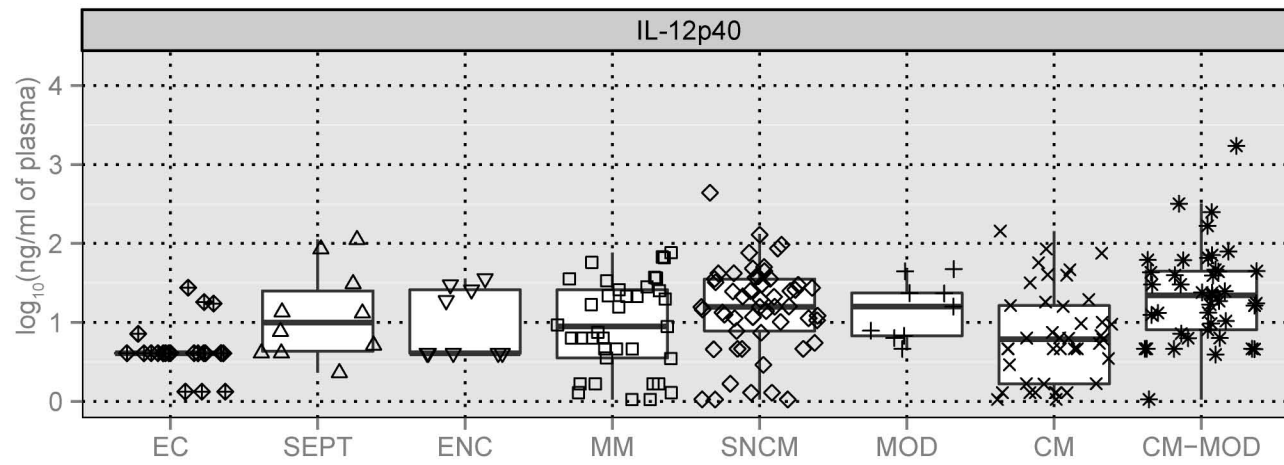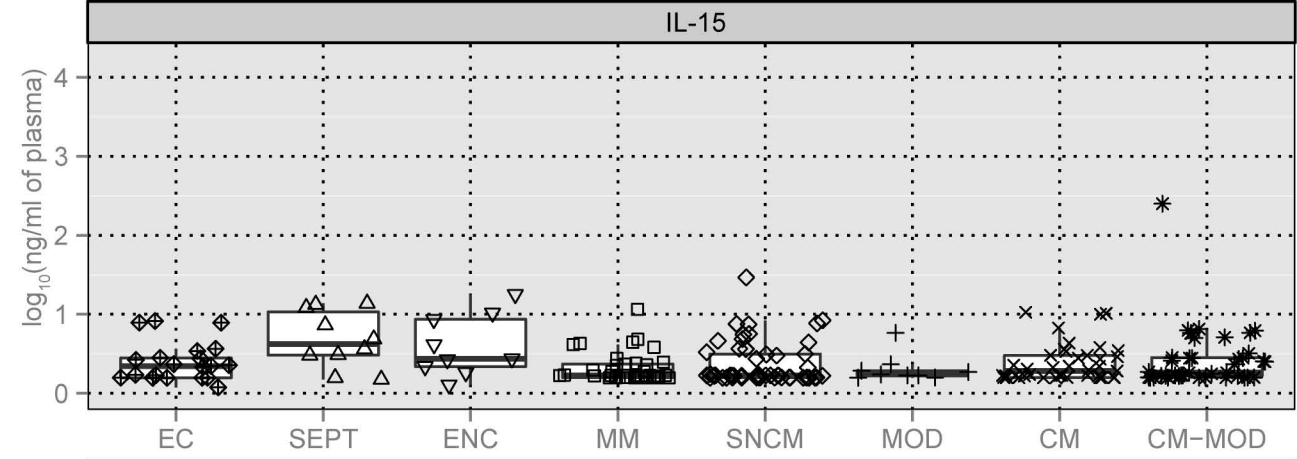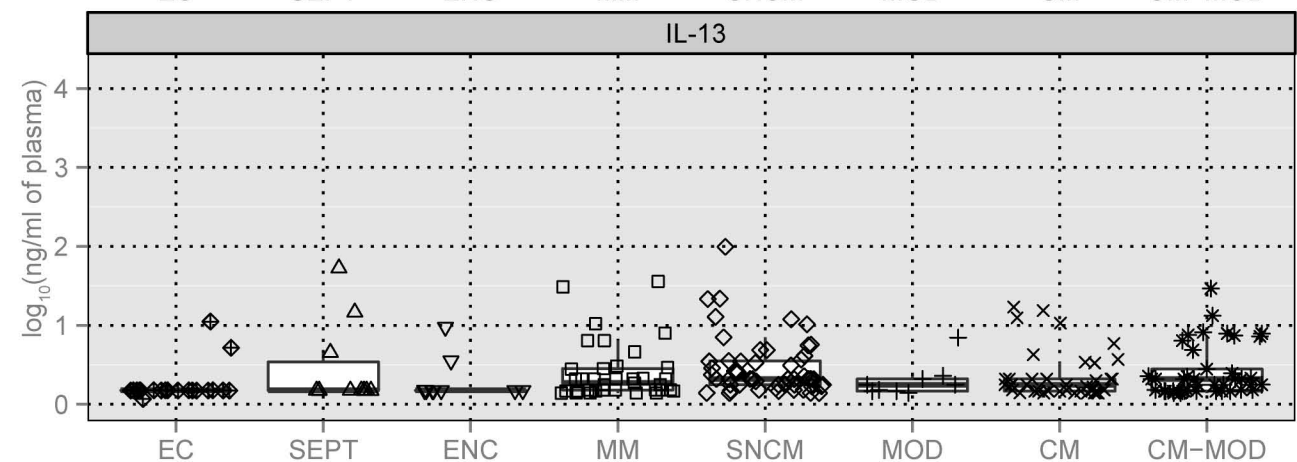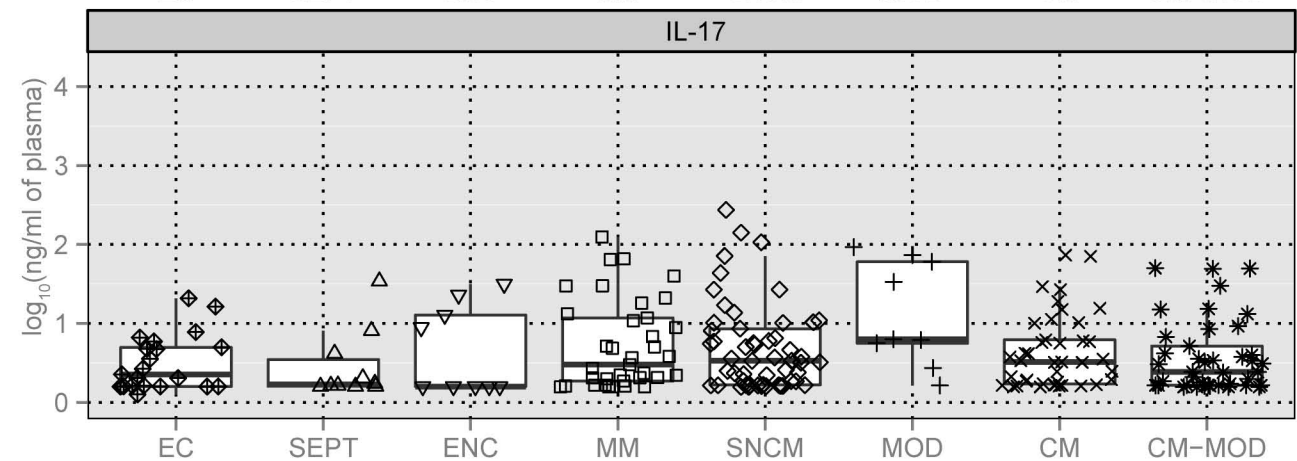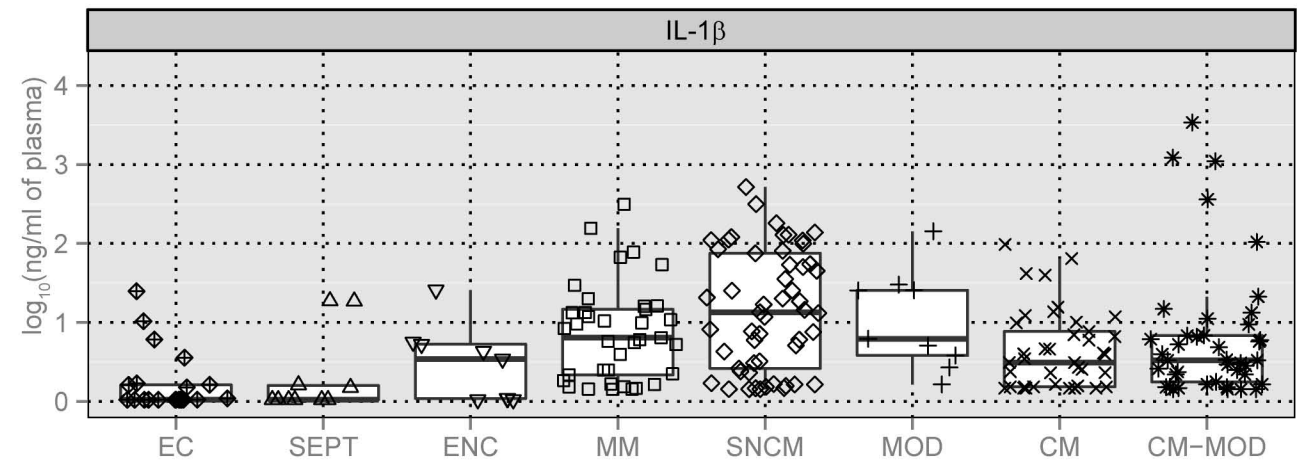

Supplement: Supplementary file 3 — 10.1186/s12967-015-0731-6 Set of cytokines characterized by relatively lower levels of expression during malaria. Boxplots and plot-jittering representations showing cytokine distributions across different subpehnotypes of malarial patients and controls. Box plots show the first and third quartiles, together with the medians of the cytokines levels expressed as log10-transformed values (ng/mL of plasma) across categories of malarial patients (MM, SNCM, MOD, CM, CM-MOD) and controls (EC, SEPT, ENC). Individual cytokines within each category are represented using a jittering method in order to reduce display overlap. [file 12967_2015_731_MOESM3_ESM.pdf]

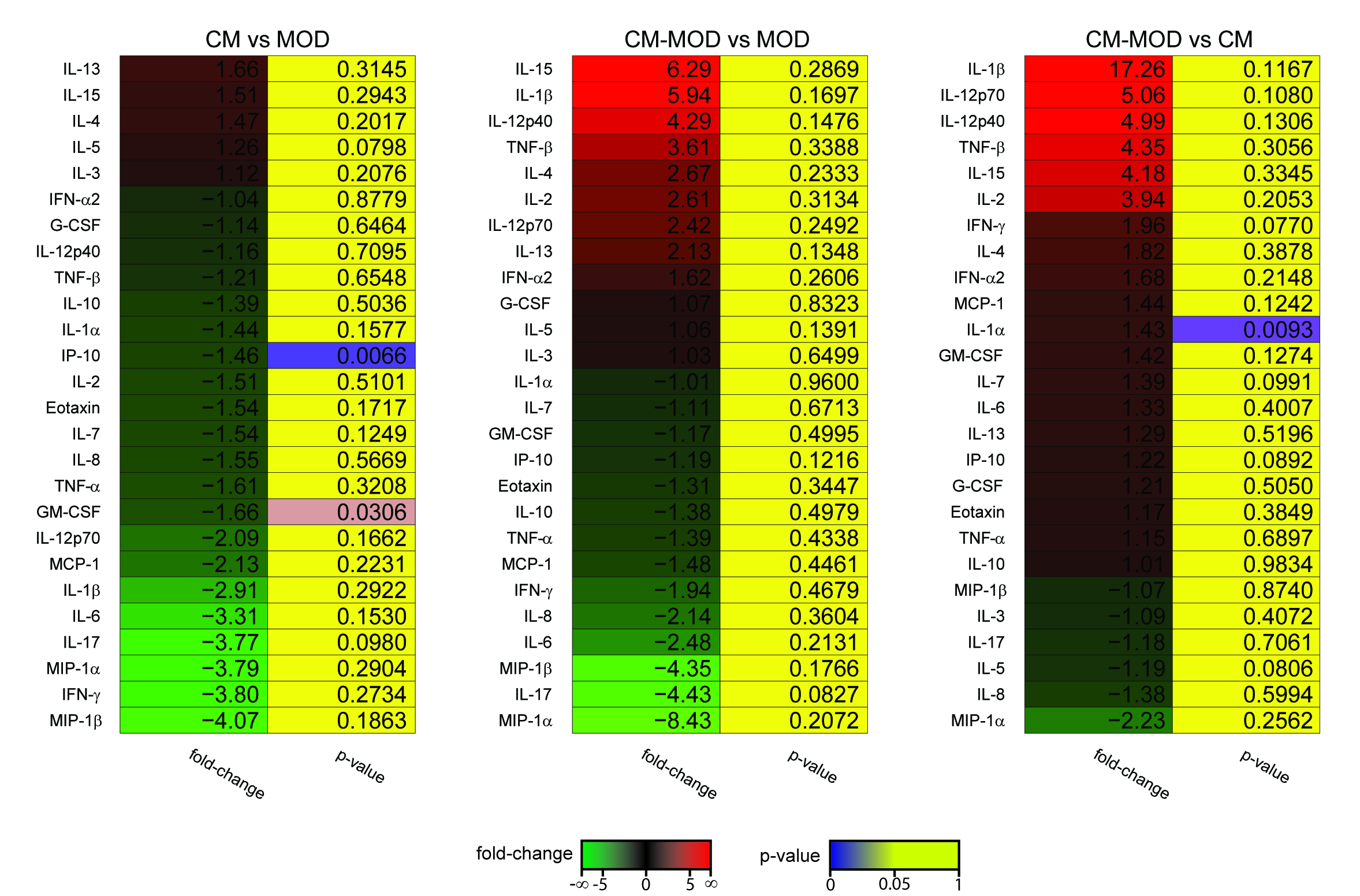

Supplement: Supplementary file 4 — 10.1186/s12967-015-0731-6 Heatmaps of cytokine signatures between MOD, CM, and CM-MOD groups. Heatmaps show the cytokine fold-change with the associated p-values for comparison between CM and MOD, CM-MOD and MOD, and CM-MOD and CM groups. Each heatmap has been sorted by the fold-change values. Fold-change values and p-values are indicated for all cytokines. [file 12967_2015_731_MOESM4_ESM.tiff]
