# Supplementary material for: Evidence of IL-17, IP-10, and IL-10 involvement in multiple-organ dysfunction and IL-17 pathway in acute renal failure associated to Plasmodium falciparum malaria
Source: J Transl Med. 2015 Nov 24;13:369. doi: 10.1186/s12967-015-0731-6 (PMC4658812; doi:10.1186/s12967-015-0731-6)
Supplement: Supplementary file 5 — 10.1186/s12967-015-0731-6 Discriminant cytokines distinguishing controls and malaria groups. [file 12967_2015_731_MOESM5_ESM.docx]

**Supplementary Table 1. Discriminant cytokines distinguishing controls and malaria groups**

| **Cytokines** | **Groups median [q1 ; q3]** | | **Ln(OR)^#^** | **p-values** |
| --- | --- | --- | --- | --- |
|  | **EC** | **Malaria** |  |  |
| **IL-4** | 0.43 [0.43 ; 0.53] | 4.01 [3.22 ; 8.37] | 66.38 | 0.0007 |
| **IL-10** | 4.08 [2.20 ; 8.69] | 264.36 [91.29 ; 863.32] | 3.26 | 0.0076 |
| **Eotaxin** | 234.96 [50.06 ; 409.64] | 159.78 [83.77 ; 265.29] | 0.15 | 0.0388 |
|  |  |  |  |  |
| **Cytokines** | **Groups median [q1 ; q3]** | | **Ln(OR)** | **p-values** |
|  | **MM** | **MOD + SNCM** |  |  |
| **IL-7** | 16.72 [7.84 ; 40.78] | 32.44 [14.29 ; 59.42] | 1.71 | 0.0112 |
|  |  |  |  |  |
| **Cytokines** | **Groups median [q1 ; q3]** | | **Ln(OR)** | **p-values** |
|  | **MM** | **CM + CM-MOD** |  |  |
| **MCP-1** | 281.20 [161.07 ; 629.90] | 578.28 [322.64 ; 1039.06] | 1.78 | 0.0053 |
| **MIP-1α** | 92.42 [29.71 ; 1251.47] | 53.58 [30.18 ; 168.54] | 0.69 | 0.0020 |
|  |  |  |  |  |
| **Cytokines** | **Groups median [q1 ; q3]** | | **Ln(OR)** | **p-values** |
|  | **MOD** | **SNCM** |  |  |
| **IL-17** | 5.30 [4.61 ; 59.32] | 2.37 [0.67 ; 7.55] | 0.62 | 0.0443 |
| **IP-10** | 9307.78 [6256.92 ; 9648.60] | 4296 [1580.30 ; 8515.69] | 0.08 | 0.0373 |
|  |  |  |  |  |
| **Cytokines** | **Groups median [q1 ; q3]** | | **Ln(OR)** | **p-values** |
|  | **SNCM + MOD** | **CM + CM-MOD** |  |  |
| **MIP-1α** | 79.84 [26.42 ; 1071.65] | 53.58 [30.18 ; 168.54] | 0.71 | 0.0002 |
|  |  |  |  |  |
| **Cytokines** | **Groups median [q1 ; q3]** | | **Ln(OR)** | **p-values** |
|  | **MOD** | **CM** |  |  |
| **GM-CSF** | 286.08 [257.48 ; 463.00] | 168.71 [105.87 ; 283.03] | 0.20 | 0.0315 |
| **IL-17** | 5.30 [4.61 ; 59.32] | 2.23 [0.65 ; 5.17] | 0.50 | 0.0263 |
|  |  |  |  |  |
| **Cytokines** | **Groups median [q1 ; q3]** | | **Ln(OR)** | **p-values** |
|  | **MOD** | **CM-MOD** |  |  |
| **IL-17** | 5.30 [4.61 ; 59.32] | 1.74 [0.65 ; 4.56] | 0.54 | 0.0303 |
| **MIP-1α** | 240.41 [54.49 ; 1619.08] | 53.33 [35.93 ; 122.69] | 0.56 | 0.0459 |
|  |  |  |  |  |
| **Cytokines** | **Groups median [q1 ; q3]** | | **Ln(OR)** | **p-values** |
|  | **MOD** | **CM + CM-MOD** |  |  |
| **IL17** | 5.30 [4.61 ; 59.32] | 2.15 [0.65 ; 5.17] | 0.53 | 0.0211 |
| **MIP-1β** | 219.74 [170.86 ; 1136.23] | 112.52 [66.23 ; 364.32] | 0.51 | 0.0238 |
|  |  |  |  |  |
| **Cytokines** | **Groups median [q1 ; q3]** | | **Ln(OR)** | **p-values** |
|  | **CM** | **CM-MOD** |  |  |
| **IL-12p40** | 5.23 [0.98 ; 15.26] | 21.86 [6.86 ; 44.20] | 1.65 | 0.0011 |

^#^ Odds ratio are in logarithm scale. q1 and q3 corresponds to 1^st^ and 3^rd^ quartiles.
