# Supplementary material for: Evidence of IL-17, IP-10, and IL-10 involvement in multiple-organ dysfunction and IL-17 pathway in acute renal failure associated to Plasmodium falciparum malaria
Source: J Transl Med. 2015 Nov 24;13:369. doi: 10.1186/s12967-015-0731-6 (PMC4658812; doi:10.1186/s12967-015-0731-6)
Supplement: Supplementary file 6 — 10.1186/s12967-015-0731-6 Univariate significance of cytokines that differentiates malarial sub phenotypes. [file 12967_2015_731_MOESM6_ESM.docx]

**Supplementary Table 2. Univariate significance of cytokines (p-values) that differentiates malarial subphenotypes.**

Significant cytokines (p-values with Bonferroni correction) that differentiate malaria subgroups are in indicated in bold. Cytokines are ranked by increasing p-values.
